# Delivery of Growth Factors to Enhance Bone Repair

**DOI:** 10.3390/bioengineering10111252

**Published:** 2023-10-26

**Authors:** Jacob R. Ball, Tara Shelby, Fergui Hernandez, Cory K. Mayfield, Jay R. Lieberman

**Affiliations:** Department of Orthopaedic Surgery, University of Southern California Keck School of Medicine, 1500 San Pablo St., Los Angeles, CA 90033, USA

**Keywords:** stem cell, gene therapy, critical-sized defect, growth factor, BMP

## Abstract

The management of critical-sized bone defects caused by nonunion, trauma, infection, malignancy, pseudoarthrosis, and osteolysis poses complex reconstruction challenges for orthopedic surgeons. Current treatment modalities, including autograft, allograft, and distraction osteogenesis, are insufficient for the diverse range of pathology encountered in clinical practice, with significant complications associated with each. Therefore, there is significant interest in the development of delivery vehicles for growth factors to aid in bone repair in these settings. This article reviews innovative strategies for the management of critical-sized bone loss, including novel scaffolds designed for controlled release of rhBMP, bioengineered extracellular vesicles for delivery of intracellular signaling molecules, and advances in regional gene therapy for sustained signaling strategies. Improvement in the delivery of growth factors to areas of significant bone loss has the potential to revolutionize current treatment for this complex clinical challenge.

## 1. Introduction

### 1.1. Bone Loss Challenge

**Figure 1:**
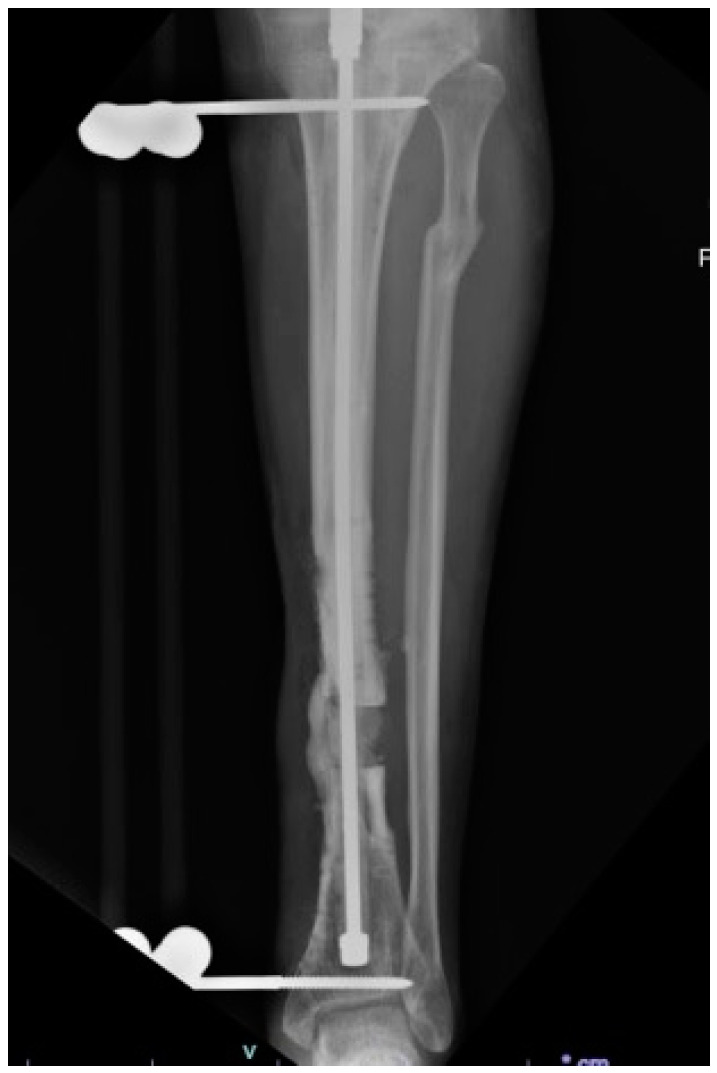
Radiograph of a tibial critical-sized bone defect associated with a fracture nonunion complicated by infection. The patient status is post-placement of an external fixator with an intramedullary antibiotic device pending distraction osteogenesis (Radiographs courtesy of Dr Joseph Patterson).

Critical-sized bone defects (Figure 1) caused by nonunion, trauma, infection, malignancy, spine pseudoarthrosis, and osteolysis pose complex reconstructive challenges for orthopedic surgeons. Failure to heal bone defects is multifactorial, with potential complications occurring at each stage of healing [1]. To achieve successful bone regeneration, there are four essential elements: (1) release of growth factors to recruit osteoprogenitor cells, (2) responding cells that mobilize to the defect, (3) osteoconductive matrix to support bone formation, and (4) a sufficient vascular supply [2]. Multiple strategies have been developed to address impairments in bone healing, including the use of autograft, allograft, growth factors, and distraction osteogenesis for larger defects [3,4,5,6,7]. Over 500,000 bone grafting procedures occur annually in the United States, with bone graft substitutes costing upwards of USD 3000 per application, resulting in USD 5 billion in annual healthcare expenditures [8,9,10,11]. Despite advances in technology and surgical techniques, significant challenges remain, including donor site morbidity for autograft harvest, insufficient graft availability, pseudoarthrosis or persistent nonunion, and prolonged immobilization in distraction osteogenesis.

### 1.2. Growth Factors for Treatment

Currently, there is significant interest in the use of growth factors to address these complex reconstructive challenges. Dating back to the 19th century, demineralized bone matrix (DBM) has been used as both xenograft and allograft for the treatment of large bone defects with mixed success [8,12,13,14]. It would take almost a century before the landmark study by Urist in 1965, where he inferred that “autoinduction” of host cell populations was responsible for the ectopic bone formation seen with implantation of allogeneic DBM [15]. This theorized inductive substance was termed bone morphogenetic protein (BMP), although it was not isolated at the time [16]. Reddi and Anderson further elucidated this pathway of DBM function, surmising there must exist some factor within the “soluble extract” allowing for the observed ectopic ossification [17]. In 1988, Muthukumaran and Reddi isolated an “osteogenin-enriched fraction” from DMB samples and demonstrated in a dose-dependent fashion that the combination of the extract with inactive collagenous bone matrix resulted in ectopic bone formation [18]. Although the exact composition of this extract was not yet elucidated, this would lead to the characterization of the now well-described family of growth factors, Bone Morphogenetic Proteins [12]. Wozney et al. further characterized BMPs by successfully preparing human complementary DNA (cDNA) from protein extracts, which allowed for the study of recombinant BMPs’ individual biologic activity as well as the development of gene therapy applications [19,20].

### 1.3. FDA-Approved Growth Factors for Bone Repair

Secondary bone repair is an intricate and dynamic cascade of molecular and cellular processes within the bone tissue microenvironment that occurs in four phases: (1) hematoma formation, (2) fibrocartilage callous development, (3) hard callus formation, and (4) remodeling [21]. These stages are highly regulated by inflammatory cytokines that temporally mediate growth factor release [22]. Tissue engineering (TE) strategies take advantage of this pathway by selecting a variety of potential growth factors that can be targeted to promote healing. Recombinant human BMP-2 (rhBMP) and platelet-derived growth factor (PDGF) are currently the only FDA-approved therapies for bone healing utilizing growth factors [23]. However, their indications remain narrow, with rhBMP-2 being FDA-approved for acute open tibial shaft fracture, alveolar ridge defects, and anterior lumbar spine surgery and PDGF for ankle and hindfoot fusions [24]. Although PGDF has shown promise in multiple clinical trials for hindfoot fusion in the setting of end-stage osteoarthritis, poor osteoinductive potential limits its application for the treatment of critical-sized bone defects [25,26,27,28]. Several other growth factors can influence the bone repair process, including vascular endothelial growth factor (VEGF) [29,30,31], insulin-like growth factor 1 (IGF-1) [32,33], fibroblast growth factor (FGF) [34,35,36], and transforming growth factor beta (TGF-β) [37]. Although these factors have established roles in bone regeneration, BMPs have consistently demonstrated their centrality to the process through in vitro assays, in vivo preclinical models, human disease, and the development of clinically important therapeutic agents [38,39]. Furthermore, BMPs alone are sufficiently osteoinductive for bone repair, which makes them ideal candidates for tissue engineering applications [40].

To promote optimal bone formation, delivery of growth factors needs to occur in sufficient quantities at the site of interest and at the appropriate time with responding cells available. Multiple preclinical TE strategies have been developed to allow for the osteoinductive signals of prospective growth factors to be paired with osteoconductive scaffolds and cells that can respond to these molecular cascades. In this review, we will highlight delivery options for growth factors, including novel scaffolds for controlled release of rhBMP, bioengineered vesicles for delivery of intracellular signaling molecules, and advances in regional gene therapy for sustained signaling strategies, as shown in Table 1.

## 2. Carriers of Bone Morphogenetic Protein and Other Growth Factors

BMPs are a family of ligands with at least 30 different members to date. Structurally, BMP homodimers include seven cysteine amino acids assisting in the formation of disulfide bonds as well as with dimerization into its biologically active form [41,42,43]. The canonical pathway of BMP signaling involves binding to cell surface receptors, leading to a complex formation composed of two dimers of type I and II serine/threonine kinase receptors, eventually resulting in activation of the Smad pathway [44,45]. Historically, BMP-2 and BMP-7 have been well described for their roles in osteoinduction. BMP-2, existing during skeletal embryonic development, has been shown as an essential component for bone fracture healing. Similarly, BMP-7 also serves a role in the fetal stage, additionally serving as an anti-inflammatory growth factor that elevates alkaline phosphatase (ALP) activity [44].

**Figure 2:**
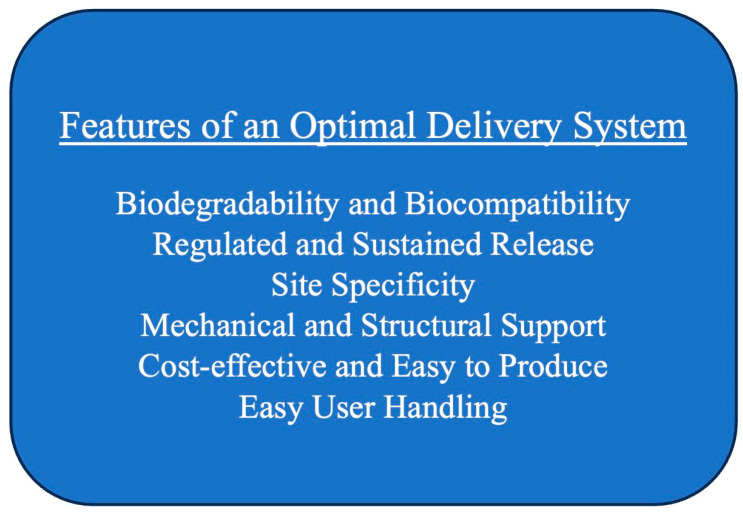
Features of an optimized delivery system.

Cloned rhBMPs are used commercially as an alternative to allograft. The FDA has approved rhBMP-2 for the treatment of open tibial shaft fractures, anterior lumbar interbody fusion, alveolar ridge augmentation, and sinus lift, as shown in Table 2 [24]. Current FDA-approved rhBMP-2 is delivered on an absorbable collagen sponge (ACS) that can be applied directly to the targeted region [46]. Alternative growth factors have also been investigated in preclinical models using an ACS for delivery, as discussed in Section 2.1. Additionally, rhBMP-7 applied in a putty composed of bovine collagen and carboxymethylcellulose for long bone nonunion and posterolateral lumbar fusion had previously received a Humanitarian Device Exemption but has since been removed from the United States market by its supplier, as it was no longer considered “medically necessary” [24]. Despite these approvals, there is concern regarding the large doses of rhBMP-2 currently used for treatment to enhance its biological activity. Not only expensive, high doses of rhBMP-2 have led to a variety of complications, including heterotopic ossification, clinically significant dysphasia associated with soft tissue edema, and inflammatory reactions [47,48,49,50,51,52,53]. Furthermore, it has been hypothesized that the rapid release of the BMP from the collagen sponge limits the body’s response [54,55,56]. Although the collagen sponge remains the only FDA-approved carrier for rhBMP-2, there is much room for improvement in next-generation carriers (Figure 2). The most common new drug delivery modalities being developed are scaffolds, broadly defined as structures that can support cell growth; these scaffold designs include porous 3D matrices, hydrogels, spheres, and fiber meshes. There are four primary categories of biomaterials used for scaffolds, including natural polymers, inorganic materials, synthetic polymers, and composites [57,58]. Despite the abundance of carriers proposed in the literature, there are significant barriers to bringing new devices to market. Clinical application of next-generation scaffolds will require significant investment and large animal models demonstrating safety and efficacy greater than current treatment modalities. Although significant challenges remain, advancement in scaffold design and composition offers promise in reducing supraphysiologic dosing requirements for growth factors in clinical practice.

### 2.1. Collagen Polymers

The prevalent connective tissue protein collagen has been well described in wound repair. The efficacy of collagen applied through absorbable sponges fabricated by freeze-drying has been extensively studied in large clinical trials. The BESTT clinical trial performed by Govender et al. demonstrated in a prospective randomized controlled trial of 450 patients with open tibia fractures that rhBMP-2 loaded ACS had significantly fewer hardware failures, infections, and faster wound healing than the control group of intramedullary nail fixation alone [59]. INFUSE^®^ (Medtronic, Minneapolis, MN, USA), a rhBMP-2 loaded ACS, is currently approved by the FDA for human use. However, despite such results, collagen sponges have demonstrated rapid collagen fiber degradation by collagenase enzymes, lack of mechanical strength, and absence of sustained release with less than 5% of the protein noted at two weeks post-implantation [58,60].

Burkus et al. performed a multi-center prospective randomized trial of 279 patients with degenerative disc disease who underwent anterior lumbar interbody fusion (ALIF) with or without the addition of rhBMP on an ACS [61]. At two-year follow-up, the experimental group had a significantly higher fusion rate (94.5%) than the control (88.7%), with similar patient-reported outcomes [61]. Additionally, radiographic healing was assessed at 6, 12, and 24 months in 42 patients, demonstrating osteogenesis in the disc space at 6 months postoperatively in 82% of patients, with most growth occurring between 6 and 12 months post-surgery [62].

Several applications using collagen sponges as carriers for growth factors other than rhBMP-2 have also been described. Kigami et al. treated a 5 mm rat calvarial defect model with an FGF-2 loaded ACS, demonstrating increased blood vessel and bone growth when compared to the ACS-only group [63]. The higher dose group of 0.3% FGF-2 showed significantly higher values in both outcomes when compared to the 0.1% FGF-2 cohort [63]. Additionally, Ueda et al. utilized TGF-β loaded collagen sponges to repair 6 mm skull defects in rabbits and demonstrated significantly more bone formation after six weeks when compared to ACS alone and free TGF-β [64]. However, it must be noted that a calvarial bone defect model is not as biologically stringent as a critical-sized long bone defect.

Several studies have been conducted to characterize the pharmacokinetics of growth factor release from collagen sponges. The BMP-2 ACS complex specifically is susceptible to significant proteolysis early postoperatively, leading to rapid elimination. Enhancements have been made to the classic collagen structure through cross-linking, carbodiimide additives, gamma radiation, and the addition of synthetic or inorganic compounds to increase stability [65]. Specifically, composites with collagen/poly(DL-lactic acid) have demonstrated improvements to the GF delivery system in preclinical models [58,66]. In a 2 cm canine ulna critical-sized defect model, Itoh et al. demonstrated significant bone formation in the experimental groups of 140 ug and 640 ug of rhBMP-2 placed on composite scaffolds [67]. Furthermore, both high and low-dose cohorts enhanced bony union with higher bone mineral content when compared to the PLGA/gelatin sponge alone [68]. Collagen additives such as heparin and fibronectin have also been described to enhance BMP binding and, therefore, lower the therapeutic dose by extending release. Lee et al. described the addition of BMP-2 on an absorbable collagen sponge with heparin sulfate nanofibers in a 5 mm rat femoral critical-sized defect model, finding that lower doses of BMP-2 generated more new bone on histological evaluation as compared to the conventional collagen sponge [69]. Interestingly, BMP contains heparin-binding domains that interact noncovalently with the heparin sulfate nanofibers, therefore prolonging release. Although these advances are promising, to our knowledge, these types of collagen sponges have not yet been FDA-approved to be used as carriers for rhBMP-2.

Gelatin, a form of type I collagen, can be formulated into hydrogels, which serve as an alternative carrier [58]. Other natural polymers that can be synthesized into hydrogels include hyaluronic acid, fibrin, or synthetic polymers such as polyethylene glycol (PEG). The mechanism of action includes entrapment using linkage molecules or with chemical makeup selective to the drug itself. Gel components can be modified to alter release through modifications of hydrogel interactions, fluid dynamics, and target tissue physiologic conditions. Hyaluronic acid is a commonly used material to formulate hydrogels. Electrostatic interactions between BMP-2 and hyaluronan are believed to mediate sustained-release kinetics, therefore prolonging the osteogenic signal [70]. In a 2 × 4 cm minipig cranial defect model, Docherty-Skogh et al. demonstrated a 100% increase in bone volume with the addition of 1.25 mg BMP-2 to an injectable hyaluronan-based hydrogel when compared to hydrogel alone [70]. Hydrogel formulations using other growth factors exist as well. In a model using FGF loaded on a p(HEMA-co-VP) hydrogel polymer scaffold, Mabilleau et al. used 4 × 6 mm cylindrical femoral condyle defects in rabbits to demonstrate increased woven bone and trabecular thickness [71]. However, this difference in bone generation became negligent between the FGF-hydrogel and the hydrogel-alone groups after three months [71].

### 2.2. Ceramics, Composites, and Applications of 3D Printing

Calcium phosphates formulated as hydroxyapatite (HA), coralline hydroxyapatite, and tricalcium phosphate (TCP) are additional materials used in scaffold development. They offer biocompatibility and the ability to withstand stress and tolerate high mechanical loads; however, drawbacks include brittleness and difficulty in generating highly porous structures needed for bony ingrowth and angiogenesis [58]. Additionally, while osteoconductive, ceramics such as HA lack osteoinductive properties. Calcium phosphates can interact with growth factors through hydrogen bonding, allowing manipulation of release kinetics through structural modifications of the carrier [72].

HA scaffolds used to deliver rhBMP-2 have demonstrated significant bone formation in a variety of settings, including alveolar ridge reconstruction, maxillary sinus augmentation, and segmental bone defects [57]. Utilizing a 3 cm sheep tibia critical-sized defect model, Boer and colleagues demonstrated 2–3 times increased torsional strength of BMP-2 loaded on HA as compared to HA alone [73]. The scaffold was created from bovine cancellous bone processed with a sintering method. Furthermore, using a 6–8 cm fibular critical-sized defect model in primates, Seeherman and colleagues showed treatment with percutaneous injection of rhBMP-2 integrated into a calcium phosphate paste resulted in accelerated healing of 40% compared with untreated sites, with torsional stiffness and maximum torque equal to an intact fibula after 14 weeks [74]. In a rat posterolateral fusion model, hydroxyapatite in the form of a fibrous mesh loaded with rhBMP-2 demonstrated a 60% greater fusion rate when compared to HA alone [75].

To enhance the osteoconductive properties of the scaffold, ceramics have historically been incorporated into composites with natural and synthetic polymers, lending it rigidity and hardness [67,76]. FDA-approved rhPDGF has successfully utilized a TCP-collagen compositive throughout clinical trials to enhance hindfoot arthrodesis [26,27,28]. Furthermore, a combination of collagen and HA has been described to create a material that simulates real bone morphology. Itoh et al. described an HA–collagen composite as a carrier for rhBMP-2 that, after 12 weeks, displayed increased bone mineral density in canine radius, ulna, and tibia defect models [54,68,77]. In another composite formulation, Chen and colleagues demonstrated that gelatin, chitosan, and HA scaffold manufactured through electrospinning and loaded with BMP-2 and VEGF led to accelerated osteogenic and angiogenic growth in a 15 mm rat calvarial defect [78].

Using an FGF-2/calcium phosphate composite layer on HA-ceramic buttons, Tsurushima et al. demonstrated efficacy in bone repair in a 5 mm rat cranial defect [79]. The HA-ceramic buttons were generated by sieving particles below 75 μm, therefore forming disks, which was followed by sintering. Also using FGF-2, Komaki et al. synthesized a β-tricalcium phosphate/collagen scaffold complex to repair a 5 mm rabbit tibial defect, ultimately demonstrating both mechanical and radiological healing at 12-week follow-up, with significantly greater bone formation when compared to the scaffold alone [80].

Three-dimensional printing has also been used to synthesize scaffolds using a variety of materials, most notably ceramics and composites. The printing method selected is determined by the scaffold material, pore size, and geometry [81]. Kolan et al. created a 3D-printed bioactive glass scaffold loaded with BMP-2, which was placed in a 4.6 mm rat calvarial critical-sized defect model [82]. The scaffold was 3D printed in both a traditional cubic formation and a complex biomimetic diamond structure. Interestingly, the diamond structure demonstrated more rapid bone formation as compared to the more traditional architecture. Three-dimensional printing also allows for composite materials to be formed, which may have enhanced osteoconductivity. Teotia et al. created a 3D-printed blend of poly(trimethylene carbonate) and ceramics (TCP or HA) loaded with rhBMP-2, which was placed in an 8 mm rabbit calvarial critical-sized defect [83]. The hydrogel blend used to generate the 3D scaffolds was formulated by dissolving gelatin in deionized water and then adding ceramic polymer resin. The composite scaffold demonstrated improved bony ingrowth compared to poly(trimethylene carbonate) alone, even prior to the addition of rhBMP-2. Through ongoing refinement of materials and architecture, 3D printing offers significant promise to develop highly osteoconductive scaffolds for growth factor delivery in bone repair applications.

### 2.3. Synthetic Polymers

Synthetic polymers have garnered attention within recent decades as effective delivery systems as the ability to synthesize specific properties allows for control over the drug release pathway. In addition to being easily reproduced, synthetic polymers such as polylactide (PLA), polyglycolide (PLG) and copolymer poly(d,l-lactide-co-glycolide) (PLGA) and PLLA are biodegradable and avoid the immunogenicity risk associated with animal-based biomaterials [54]. Although much research has been directed towards 3D porous scaffolds, other formulations of synthetic polymers have been pursued, such as microspheres, fibers, and sheets [54]. Using a 7.9 mm rabbit calvarial defect model treated with BMP-2 incorporated in PLGA microspheres suspended in carboxymethylcellulose (CMC), Woo et al. created immediate and sustained-release preparations of growth factor by altering the physical properties of the microspheres [54]. PLGA microspheres were synthesized through a double emulsion technique. Sustained-release microspheres had detectable BMP-2 release at 21 days post-implantation compared to 7 days in the immediate-release implants. Additionally, the prolonged-release implant exhibited 75–79% calvarial defect healing compared to the 45% in the immediate-release cohort at 6 weeks post-implantation [54]. In a rat calvarial defect model, Weber et al. demonstrated that rhBMP-2 PLGA microspheres increased bone thickness by almost 100% compared to a rhBMP-2 gelatin-hydrogel cohort [77].

As discussed previously, altering a scaffold’s biochemical makeup can allow for tighter control over its release kinetics. There has been significant interest in creating scaffolds that respond to stimuli such as pH change, temperature, or local enzymatic degradation. In regions of local acidosis, substrates with pH sensitivity, such as poly(acrylic acid), allow for the facilitation of drug delivery. Various polymers combine this pH sensitivity with temperature dependency as well, allowing for phase transitions at physiologic temperatures [84,85,86,87,88,89,90,91]. One such polymer (PCLA-PEG-PCLA) was altered by Kim and colleagues to include thermo- and pH-sensitive components and injected into the dorsum of mice [92]. The thermos- and pH-sensitive polymer demonstrated a phase transition from suspension to gel In vivo, with high encapsulation efficiencies of rhBMP-2 and human mesenchymal stem cells with bone tissue formation after 7 weeks [92]. Additionally, multiple studies have demonstrated that the incorporation of molecules with proteolytic sensitivity, such as susceptibility to matrix metalloprotease (MMP) and/or plasmin, can be developed as novel controlled-release scaffolds [90,93,94,95,96].

Temporal associations between different growth factors have also been assessed. Utilizing a composite scaffold of PPF/gelatin + PLGA microparticles, Kempen et al. designed a temporal system of delivering both VEGF and BMP-2 [97]. PLGA microspheres, generated through double emulsion, were formulated into a composite with PPF through photo-crosslinking. Interestingly, there was significantly greater ectopic bone formation in the combined growth factor treatment group than in the scaffold and BMP-2 alone, with the authors deducing the benefit of sequential angiogenic and osteogenic signals in bone formation [97]. This dual release and synergistic effect of VEGF and BMP-2 in early bone generation was further studied using a porous poly(propylene fumarate) scaffold to repair 5 mm rat femoral defects, resulting at four weeks in significantly higher ectopic bone formation than BMP only, VEGF only, or empty scaffolds [97]. Lastly, Sharmin et al. developed a polymer-coated allograft that sequentially releases VEGF followed by BMP-2 in an attempt to replicate physiologic growth factor cascades [98]. Differential release kinetics were hypothesized to be a function of molecular weight differences between VEGF and BMP-2, resulting in prolonged entrapment of VEGF in surface pores. In a 6 mm rat femoral critical-sized defect model, the VEGF/BMP-2 loaded polymer-coated allograft exhibited significant bone growth from four to eight weeks compared to BMP-2 alone polymer-coated allograft, again suggesting that temporal release of growth factors may enhance osteogenesis [99].

## 3. Extracellular Vesicles

Extracellular vesicles (EVs) are double phospholipid bilayer nanoparticles that are released by most cell types to mediate cell-to-cell communication [100]. Historically, they have been categorized by size and biogenesis: apoptotic bodies secreted by dying cells and exosomes secreted by viable cells through outward budding of the plasma membrane [101]. They are naturally occurring biological transporters that carry lipids, proteins, nucleic acids, and other bioactive molecules [102]. Additionally, EVs can serve as carriers of growth factors and downstream signaling molecules that have been shown to activate cellular cascades that promote bone remodeling through increased osteoblast proliferation, angiogenesis, and immunoregulation [103]. Their phospholipid bilayer facilitates these functions by acting as a barrier against the rapid clearance of cargo, protecting against enzymatic degradation, and facilitating the crossing of biological membranes [104,105]. For these reasons, there has been an extensive preclinical evaluation of EVs as delivery vehicles of growth factors and downstream signaling molecules as a novel strategy to repair critical-sized defects [100].

### 3.1. Role of Extracellular Vesicles in Growth Factor Delivery and Bone Repair

**Figure 3:**
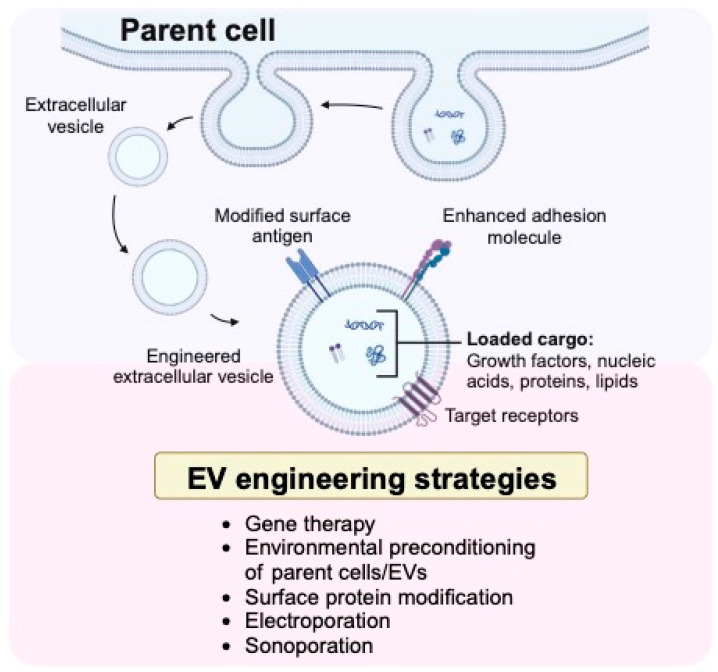
Schematic illustration of a parent cell undergoing ectocytosis of an extracellular vesicle and the engineering strategies to optimize extracellular vesicles (EVs) for bone repair (The figure was created with BioRender.com).

The cargo within EVs largely reflects the parent cell from which they originate [106]. The most clinically relevant cell types for derived EVs in bone tissue engineering include osteoblasts [107], osteocytes [108], and mesenchymal stem cells [109,110,111,112]. There has been mounting evidence demonstrating that EVs serve as important mediators of MSC paracrine effects through intercellular communication and transfer of bioactive molecules [100,113]. EVs present an opportunity to improve upon traditional stem cell therapy through enhanced biocompatibility and targeting of cells [113,114,115]. These therapeutic effects have been observed in preclinical models of bone regeneration, and to our knowledge, there is one registered clinical trial utilizing MSC-derived EVs to address bone defects [116]. In addition to highlighting the role of EVs and their respective cargo in bone regeneration (Table 3), we will discuss strategies for engineering EVs to optimize cargo yield and delivery of growth factors (Figure 3).

#### In Vivo Extracellular Vesicle Mediated Delivery of Growth Factors

The effects of EVs in bone regeneration are mediated by intercellular communication relayed by their cargo [118,120]. Critical-sized defect models have demonstrated the pro-osteogenic and angiogenic effects of EV cargo delivery through the transcriptional upregulation of osteocalcin (OCN), type I collagen (COL I), alkaline phosphatase (ALP), osteopontin (OPN), vascular endothelial factor (VEGF), angiopoietin 1 (ANG1) and angiopoietin 2 (ANG2) [111,126,127]. In a rat femoral nonunion model, animals treated with 10^10^ particles of bone marrow mesenchymal stem cell-derived EVs (BMMSC-EVs) had significantly increased fracture callus observed on radiographs, improved bone volume on microCT, and histologic evidence of healing on postoperative week 8 compared to non-treated controls [117]. Furthermore, PCR and Western blotting demonstrated increased gene expression of VEGF and hypoxia-inducible factor 1-alpha (HIF-1α) at the nonunion site in the experimental cohort. Additionally, Zhang et al. found an upregulation of proteins involved in the BMP-2/Smad1/RUNX pathway, a signaling cascade found to increase osteogenic differentiation and essential for fracture healing [118,120]. The upregulation of osteogenic proteins and activation of growth factor-mediated signaling cascades implies the presence of crosstalk between EV cargo and pro-osteogenic pathways important for tissue engineering applications [117].

### 3.2. Engineering Extracellular Vesicles to Optimize Growth Factor Delivery

Although naturally occurring EVs play a role in growth factor delivery, TE strategies have been developed to enhance their therapeutic impact on bone repair. Endogenous and exogenous strategies have demonstrated effectiveness in selectively loading EVs, increasing payload, and improving homing efficiency to bone defects [128,129,130,131]. Endogenous engineering is the enhancement of parent cells to produce desired EV phenotypes, while exogenous engineering is a direct manipulation of EVs. Engineering techniques to optimize EV delivery of growth factors include gene transduction, electroporation, sonication, preconditioning, surface modification, and mechanical manipulation [132,133,134]. Despite ongoing improvements in selective cargo loading, significant variability in preclinical dosing paradigms hinders clinical translation [135]. EV protein weight and particle dosing protocols inadequately assess growth factor quantity and may not be appropriate for clinical application. Although significant challenges remain, ongoing advances in EV cargo analysis offer promise in standardizing doses for the treatment of large bone defects.

#### 3.2.1. Endogenous Engineering of Parent Cells

Endogenous EV cargo loading through engineering parent cells is an attractive strategy to increase growth factor yield and delivery. Preconditioning of parent cells by environmental manipulation is one technique that has been investigated [136]. A common strategy is hypoxic preconditioning of MSCs, as this has been shown to increase the angiogenic effects of secreted EVs as HIF-1α is upregulated and stabilized in hypoxic conditions [137]. In an in vivo study utilizing this method, 100 μL of umbilical mesenchymal stem cell (uMSC) derived EVs stimulated bone healing in a rat femoral fracture model by enhancing the proliferation of human umbilical vein endothelial cells (HUVECs), which in turn, produced angiogenic growth factors [120]. Physical methods of preconditioning have also been investigated. Osteocytes are mechanosensitive cells that alter gene expression when under mechanical stress. Eichholz et al. studied the release of EVs from osteocytes under different loading environments and found that 1 μg of mechanically activated osteocyte-derived EVs (MA-Evs) upregulate histone H4, which promotes osteoblastogenesis through the inhibition of RANKL-RANK [124].

Viral/nonviral transduction (see Section 4.2 and Section 4.3) of parent cells to constitutively express osteogenic and angiogenic-related proteins is another attractive endogenous engineered alternative [122,138]. Transduced parent cells can dramatically increase the production of osteogenic- and angiogenic-related growth factors, which maximizes the therapeutic effects of their released EVs [139]. Li et al. evaluated BMMSCs transfected with an adenovirus to overexpress HIF-1α for the treatment of steroid-induced osteonecrosis of the femoral head in a rabbit model [119]. The EVs released from these cells were isolated and injected into the femoral head and compared to non-transfected control EVs. There was greater micro-vessel density and trabecular bone formation within the transfected EV group secondary to the endogenous cargo loading of growth factor [119].

#### 3.2.2. Exogenous Engineering of Extracellular Vesicles

Exogenous engineering of EVs allows for more precise cargo loading. Two strategies under investigation are electroporation and sonoporation, which cause transient permeability of the plasma membrane to facilitate loading. In an in vivo study using a rat radial defect model, a plasmid carrying VEGF was introduced into EVs derived from a chondrogenic progenitor cell line via electroporation, and 10 μg of the engineered EVs were injected into the defect site, resulting in greater bone formation as shown on microCT compared to unmodified EVs [140]. Alternative methods to enhance the homing efficiency of engineered EVs are also in development. EVs express specific surface proteins that aid in cell targeting and promote communication with their surroundings [141]. Surface modification of EVs has been shown to improve cell targeting and delivery of cargo, therefore enhancing activation of pro-osteogenic signaling cascades [142]. Luo et al. utilized an osteoporotic mouse model with a BMMSC-targeting aptamer that was conjugated to bone marrow stromal cell-derived extracellular vesicles (ST-EVs) to demonstrate enhanced ST-EV homing efficiency [143]. The animals treated with 10 μg of an intravenous injection of the modified EVs demonstrated improvement in bone mineral density compared to the nonmodified EV cohort [143]. The aptamer-conjugated ST-EVs had better accumulation in bone with increased trabecular formation when compared to unconjugated ST-EVs.

#### 3.2.3. Optimizing Extracellular Vesicles for Growth Factor Delivery

A primary concern in utilizing EVs in bone defects is their rapid enzymatic clearance. The addition of scaffolds, as discussed in Section 2.1, Section 2.2 and Section 2.3, can help maintain a local EV concentration by prolonging release [114]. The pairing of scaffolds and engineered EVs has been an area of significant interest to optimize the regenerative effects of EV cargo further. Li et al. found that human ADSC-derived EVs loaded on a PLGA/polydopamine(pDA) scaffold promoted the recruitment of endogenous MSCs to increase bone formation in a mouse 5 mm critical-sized calvarial defect when compared to the scaffold alone [100]. PLGA/pDA loaded with EVs demonstrated sustained release of EVs over eight days compared to four in unmodified PGLA, demonstrating significant potential for optimization of release kinetics [95]. As described earlier, modifications to natural polymers, synthetic polymers, hydrogels, ceramics, and composites have all been described and have been successfully implemented in sustained-release strategies for EVs to enhance the repair of bone defects [144,145,146,147].

## 4. Role of Regional Gene Therapy in Growth Factor Delivery

Delivery options for growth factors discussed in earlier sections of this review rely upon the in-situ biology of the fracture environment to provide osteogenic cells to aid in bone healing. Despite the highly regulated repair process that exists [148], critical-sized defects disrupt the environment beyond the endogenous repair capabilities, resulting in nonunion. Advances in tissue engineering over the last few decades have yielded technology that helps to address these challenges. Local delivery of nucleic acids or cells transduced with genetic sequences that express growth factors has shown promise in overcoming the obstacles posed by disrupted healing environments. The strength of regional gene therapy is that osteoinductive growth factors can be directed to the defects along with essential cell populations placed on an osteoconductive scaffold, therefore overcoming the disrupted healing process [149].

### 4.1. In Vivo Versus Ex Vivo Regional Gene Therapy

In vivo and ex vivo regional gene therapy techniques have been developed with successful application in bone regeneration. In vivo therapy involves either viral or nonviral vectors carrying genes of interest being directly administered either locally or systemically to the patient. The vector is then free to transfer genetic material to cellular populations either through specific or nonspecific binding. The strength of in vivo gene therapy is that it is performed as a single-stage procedure without the need for prior cell harvest. This has significant potential advantages over ex vivo therapy from a clinical feasibility and cost reduction perspective, but there are certain limitations associated with this strategy. Challenges with in vivo regional gene therapy include nonspecific selection of target cells, inability to place an osteoconductive scaffold, and inefficient transduction. Despite these limitations, there continues to be significant interest in the development of in vivo regional gene therapy with promising preclinical results in bone regeneration [149].

**Figure 4:**
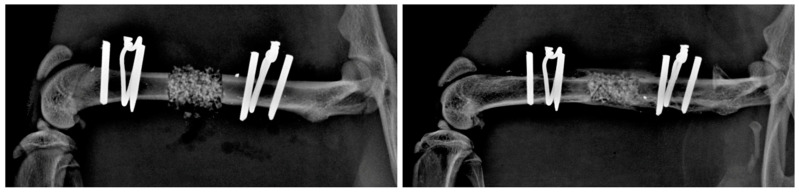
Radiograph of a 6 mm athymic rat critical-sized femoral defect treated with LV-BMP-2 transduced human adipose-derived stem cells loaded on a TCP-HA scaffold at postoperative day 0 (**left**) with the healed defect at 12 weeks postoperatively (**right**).

Ex vivo gene therapy requires harvesting autologous or allogeneic cells of interest, culturing and expanding cell populations, and finally, transducing genetic material prior to implantation at the site of interest. One of the advantages of ex vivo regional gene therapy is that a large quantity of healthy transduced cells can be prepared, which is important for healing large defects. There has been a significant effort to identify cells that can be abundantly harvested, easily expanded, consistently transduced, and highly expressed growth factors [2]. The osteogenic potential of human adipose-derived stem cells and human bone marrow-derived stem cells (BMMSC) has been evaluated in bone-healing applications [150,151]. Interestingly, lentivirus BMP-2 transduced human adipose-derived stem cells resulted in significantly higher levels of BMP-2 production in vitro and also superior healing in a rat critical-sized femoral defect model (Figure 4) [150,151]. Furthermore, adipose tissue is readily harvested through liposuction procedures rather than more invasive bone marrow harvests from the iliac crest, which is an important consideration for clinical use.

The drawback to ex vivo therapy is that autologous cells often need to be collected in staged procedures to allow time for preparation. To overcome this obstacle for clinical development, same-day techniques have been developed and proven effective [150,152]. Additionally, allogeneic cells may be feasible protein carriers, which would allow the preparation of transduced cells in advance of definitive treatments. The development of an “off-the-shelf” product would significantly improve the utility of ex vivo therapy in clinical applications.

### 4.2. Viral Vectors

**Figure 5:**
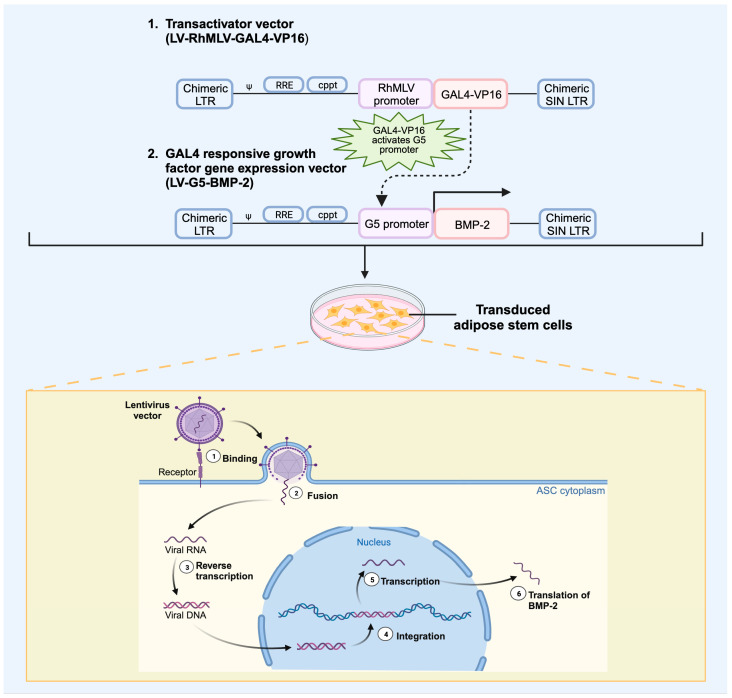
Schematic illustration of two-step transcriptional amplification (TSTA) lentiviral system overexpressing growth factor genes of interest in adipose stem cells. The TSTA system is composed of two separate lentiviral vectors: the GAL4-VP16 (LV-RhMLV-GAL4-VP16) transactivator vector and the transgene vector expressing the growth factor gene (LV-G5-GF gene) (Created with BioRender.com). LV, lentivirus; RhMLV, murine leukemia virus; LTR, long terminal repeat; Ψ, packaging signal; RRE, rev-responsible element; cppt, central polypurine tract; G5 promoter, GAL4 binding site; SIN, self-inactivating; GF, growth factor; ASC, adipose stem cell; BMP-2, bone morphogenetic protein 2.

Viral vectors have been successfully employed in regional gene therapy in preclinical applications for bone repair [20]. Viral vectors are replication incompetent viruses that have been genetically modified to carry transgenes that can be delivered to cellular populations, as shown in Figure 5. Historically, these vectors have been more efficient than nonviral vectors in transducing cells to express genes, although safety concerns include off-target transduction, severe immune response, and potential tumorigenesis [149]. Despite these challenges, viral vectors have been employed for the treatment of Wiskott–Aldrich syndrome, leukodystrophies, thalassemias, familial lipoprotein lipase deficiency, congenital amaurosis, and other genetic conditions [153,154,155,156]. Additionally, in preclinical models, viral vectors carrying genetic sequences for growth factors have been used to heal fractures and critical-sized defects as well as enhance spinal fusions. Selection of viral vectors is critical to this technique, given differences in immunogenicity, tropism, duration of gene expression, and packaging capabilities.

The first viral vectors utilized in bone tissue engineering were derivatives of adenovirus due to their low pathogenicity, high tropism, ability to carry large DNA sequences, and familiarity with oncogenic applications [157,158,159,160]. The major challenge posed by AV vectors is that they do not integrate into the host genome, which limits the duration of gene expression [161,162]. Additionally, AV vectors have significant immunogenicity, often requiring the use of immune-incompetent preclinical models [163,164,165].

Adeno-associated virus (AAV) has also been investigated as a potential vector for the delivery of growth factor in regional gene therapy for bone repair, given its reduced immunogenicity, transduction of dividing and nondividing cells, and prolonged gene expression [166]. Although AAV has proven effective in gene therapy applications, complex packaging restricts transgene size to less than 5.0 kb, which severely limits its efficiency in bone repair [167]. Additionally, in vitro studies have demonstrated poor transduction potential with significantly reduced BMP-2 production without osteogenic differentiation in multiple human stem cell lines compared to a lentiviral vector (LV) [168].

Lentivirus is a single-stranded RNA virus derived from HIV-1, which integrates into the host genome, allowing for prolonged transgene expression [169]. Furthermore, LV can infect nondividing cells and integrate into host-transcribed gene regions rather than regulatory sites, which reduces its oncogenic potential [153]. Safety modifications have been implemented for third-generation LV vectors, including the deletion of non-essential viral proteins and the separation of genomes to multiple plasmids, therefore reducing the probability of reconstitution [170,171]. LV has been successfully applied in multiple preclinical models of bone healing and spinal fusions due to its robust transduction efficacy, sustained expression of growth factor transgenes, and low immunogenicity profile [161,163,172,173].

#### Viral Vector Delivery of Growth Factor in Bone Repair

Viral vectors have shown significant promise in regional gene therapy for the delivery of growth factors in bone repair applications, as demonstrated by our lab. They have predominantly been used to transduce the BMP-2 gene, as shown in Table 4 and Table 5, but there are preclinical studies investigating their use with other growth factors as well. An early study by Lieberman et al. used an AV vector carrying a BMP-2 cDNA (Ad-BMP-2) to transfect autologous rat bone marrow cells (RBMC) implanted onto a DBM carrier [20]. The loaded carrier was placed in an 8 mm critical defect in a rat hind leg, which was successfully healed at 12 weeks postoperatively. Additionally, biomechanical properties were restored in the operated limb when compared to contralateral controls. AV vectors have also been successfully employed in spinal fusion models utilizing multiple growth factors, including BMP-2, BMP-6, BMP-7, and BMP-9 [20,174,175,176,177,178]. In these studies, AV-BMP was administered through in vivo or in vitro techniques, with successful fusion seen on radiographs and microCT demonstrating continuous contact with posterior spinal elements. Challenges with using AV to deliver growth factors have also become apparent in preclinical models. Significant inflammatory responses have been observed with the use of Ad-BMP-2, which can inhibit the production of ectopic bone; however, administration of immunosuppressive agents such as tacrolimus alongside Ad-BMP-2 may reduce the immune response and improve bone regeneration [164,165,179].

**Figure 6:**
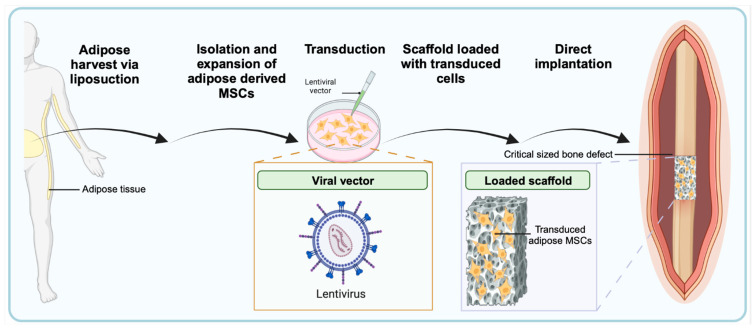
Human Adipose-Derived Stem Cells transduced with LV-BMP-2 loaded on a scaffold prior to implantation in a critical-sized bone defect. MSCs, mesenchymal stem cells; LV-BMP-2, lentivirus vector expressing bone morphogenetic protein. (The figure was created with BioRender.com).

Given the favorable tissue engineering profile for LV vectors, as discussed previously, these have been extensively employed in the delivery of growth factors in bone-healing applications. Viral integration of LV can provide sustained release of transduced BMP-2 for longer than 3 months, which has been shown to improve healing rates on XR, increase bone volume to tissue volume on microCT, and increase energy to failure on biomechanical testing when compared to AV-BMP-2 in a critical-sized rat radius defect model [161,162]. Additionally, preclinical studies have demonstrated success with transducing human-harvested cells to treat critical-sized defects in animal models. Vakhshori et al. obtained adipose tissue from elective liposuction procedures of female donors and isolated human adipose-derived stem cells (hASC), which were transduced using an LV-BMP-2 vector (Figure 6) [151]. The transduced hASCs were then loaded on a tricalcium phosphate/hydroxyapatite carrier and placed in an athymic rat 6 mm critical-sized femoral defect model. An athymic rat model was used to avoid an immune response to the hASCs. At 12 weeks postoperatively, 13 of 14 animals with LV-BMP-2 hASCs successfully healed the femoral defect, which was equivalent to treatment with rhBMP-2. Furthermore, the LV-BMP-2 hASC cohort demonstrated significantly increased bone volume on microCT, greater circumferential bone formation on histology, and improved biomechanics compared to non-transduced cohorts. The ongoing investigation of LV-transduced human-derived stem cells provides an exciting opportunity for the bench-to-bedside application of this technology.

In addition to the delivery of BMP, there are a few studies demonstrating the feasibility of regional gene therapy in the delivery of alternative growth factors in bone repair. Peng et al. prepared a retroviral vector with VEGF and BMP-4 cDNAs, which were used in combination to transduce muscle-derived stem cells loaded on a gelatin sponge to heal a mouse 6 mm calvarial defect [186]. Interestingly, combined VEGF/BMP-4 regional gene therapy enhanced bone formation but only at ratios of 1:5 and 1:1, with inhibitory effects seen at 5:1. Ito et al. demonstrated in a rat fracture model that 4 mm structural allograft coated with AAV-VEGF and AAV-RANKL could revitalize the tissue resulting in remodeling of the graft with radiographic evidence of healing [187]. Lastly, in a rat alveolar defect model, Ad-PDGF placed on a type I collagen sponge demonstrated significantly improved trabecular bone thickness, defect filling, and biomechanical properties compared to a sponge with Ad-Luciferase control [188].

### 4.3. Nonviral Vector Delivery of Growth Factor in Bone Repair

Given concerns of immunogenicity, pathogenicity, and possible tumorigenesis with the use of viral vectors, there have been significant efforts underway to develop nonviral transduction techniques. Traditionally, these techniques have resulted in low levels of transgene expression in target cells, which has limited their application in bone regeneration. Despite these challenges, preclinical applications have utilized liposomes, polymers, plasmids, sonoporation, and electroporation to deliver growth factor genes into target cell populations with varying success [189,190,191,192].

Although less frequently applied than viral vector-mediated regional gene therapy, multiple groups have demonstrated bone regeneration using nonviral methods. Sonoporation has been investigated as a potential physical delivery mechanism, given its noninvasive nature and technical reproducibility [190,191]. Bez et al. created a miniature pig 1 cm critical-sized tibial defect model and placed a collagen scaffold within the wound [190]. By postoperative day 14, the experimental group received an injection of BMP-6 plasmid mixed with microbubbles at the fracture site, followed by ultrasonic pulses until bubble oscillations were no longer visualized. At 5 days post-injection, only 40% of the cells at the defect were found to be transduced; however, there was 120-fold higher BMP-6 production compared to empty plasmid controls. At 8 weeks postoperatively, there was 75% restoration of the defect, which was similar to autograft controls with biomechanical equivalence. Feichtinger et al. also used sonoporation to transduce a plasmid containing both BMP-2 and BMP-7 genes into cells to heal a rat 4 mm femoral defect model [192]. Interestingly, passive gene transfer was found to have a 61% transduction success compared to 100% of the animals with ultrasonic pulses. Unfortunately, only 2/6 (33%) experimental animals had successful bony unions, as seen on microCT, compared to 1/6 (16.7%) with passive transduction alone.

Alternative nonviral techniques have made use of polymers to effectively deliver growth factors in preclinical models. Curtin et al. compared the effectiveness of polyethyleneimine (PEI) and nano-hydroxyapatite (nHA) vectors loaded on collagen-nHA scaffolds to heal a mouse 7 mm cranial defect by creating PEI-BMP-2/PEI-VEGF and nHA-BMP-2/nHA-VEGF [193]. Interestingly, PEI-BMP-2 and PEI-VEGF resulted in the highest mesenchymal stem cell (MSC) growth factor production; however, in vitro studies demonstrated greater calcium deposition with nHA-BMP-2 and similar endothelial proliferation with both VEGF vectors. Furthermore, nHA-BMP-2/nHA-VEGF dual therapy loaded on a hydroxyapatite scaffold resulted in the greatest healing in a mouse cranial defect model with PEI-BMP-2/PEI-VEGF dual therapy, not resulting in improvement compared to scaffold alone.

### 4.4. Role of Scaffolds in Gene Therapy

In addition to providing an osteoinductive surface for bony ingrowth, scaffold design greatly impacts the controlled delivery of growth factors in regional gene therapy. As discussed previously, scaffolds can be composed of various biomaterials and can transport not only recombinant protein but also plasmid DNA (pDNA), chemically modified RNA, and genetically modified MSCs. One area of interest is the utilization of 3D-printed scaffolds, which have the added benefit of being customized to fit any bone defect that may be encountered and clearly have clinical potential. Alluri et al. obtained preoperative microCT scans of a 24-week-old rat’s femur, which was templated for a 3D printed β-tricalcium phosphate scaffold [194]. The 3D printed scaffold was then loaded with LV-BMP-2 transduced RBMCs prior to implantation in a rat 6 mm femoral critical-sized defect. Interestingly, all rats with transduced RBMCs loaded onto the scaffold demonstrated healing at 12 weeks postoperatively. The ongoing development of 3D-printed scaffolds in regional gene therapy presents an opportunity to treat clinically complex defects that off-the-shelf products would inadequately address.

Additionally, scaffolds can be used in a nonviral context, forming what are traditionally termed gene-activated matrices (GAMs). Challenges with GAMs include low transduction efficacy in a limited population of available osteoprogenitor cells. Despite these obstacles, GAMs have been used in clinical applications of bone regeneration. Bozo et al. employed a collagen-hydroxyapatite scaffold and pDNA-VEGF complex to repair a mandibular nonunion in a 37-year-old patient with a resected fibrous dysplasia who failed multiple bone grafting procedures [195]. At 12 months postoperatively, Bozo et al. reported that there was significant integration of the GAM, healing of the defect, and remodeling of the nonunion site without observed adverse events [195]. Given this early success, a nonrandomized trial was conducted with 20 patients with maxillofacial bone defects who were treated with a GAM composed of ostacalcium-carrying pDNA-VEGF [196]. At 6 months postoperatively, all patients demonstrated significantly increased bone density and bone healing with the grafting area composed of newly formed tissue and GAM resorption.

## 5. Conclusions and Future Directions

Delivery of growth factor for repair of large bone defects is a promising solution to this complex clinical challenge. As discussed, local delivery of growth factors, extracellular vesicles, and regional gene therapy continue to be viable options for further clinical development. Significant challenges remain, including the development of osteoinductive scaffolds with sustained growth factor release, selective loading of extracellular vesicles, and optimization of gene therapy for reduced immunogenicity. A major advancement in the development of all these technologies will likely be achieved through improved scaffold design. Although natural polymers and ceramics are most prevalent in clinical practice today, improvements in synthetic polymers and signal-responsive scaffolds can dramatically improve the delivery of growth factors. Additionally, an increased understanding of extracellular vesicles and their cargo has the potential to change how stem cells are utilized in bone regeneration applications. Engineered EVs with intracellular osteoinductive cargo may allow for modulation of molecular pathways not currently achievable with local delivery of growth factor. Furthermore, an improved understanding of biologically critical pathways for bone regeneration, such as SMAD, WNT, Notch, and Hedge Hodge, may provide alternative therapeutic targets [197]. Emerging technology such as CRISPR and the use of miRNA may greatly improve our ability to target cellular pathways through modification of parent cells [198,199,200,201]. Finally, regional gene therapy has significant clinical potential. The combination of locally produced osteoinductive signaling generated through selected cellular populations placed on an osteoconductive scaffold has the potential to revolutionize how these bone defects are treated in clinical settings [198,199,200,201]. Ongoing development of ex vivo allogeneic regional gene therapy provides an exciting opportunity to develop an “off-the-shelf” therapeutic to treat large bone defects [202,203].

## Figures and Tables

**Table 1:** Summary of growth factor delivery strategies in bone repair.

Delivery Method	Advantages	Challenges
Scaffolds	- Local delivery of purified growth factors with precise concentrations - Modifiable composition/geometry to improve osteoconductivity - Customizable for complex defects - Potential for time-dependent release	- Biocompatibility dependent upon material - Sustained release requires significant modifications - Complex manufacturing
Extracellular Vesicles	- Immune privileged - Enhanced crossing of biological membranes for intracellular signaling - Modifiable for homing efficiency - Protection against enzymatic degradation	- Isolation and collection - Nonspecific cargo loading and concentrations - Undefined signaling pathways
Regional Gene Therapy	- Sustained signaling with viral integration - Pairs osteoinductive signaling with responding cells and osteoconductive scaffold	- Potential inflammatory response dependent upon vector - Limited concerns for viral reconstitution and oncogenesis - Complex manufacturing

**Table 2:** FDA-approved clinical applications of recombinant BMP-2.

Systems	Indication	Approval Date
INFUSE^®^ Bone Graft	Alternative to autogenous bone graft for sinus augmentations For localized alveolar ridge augmentations in extraction socket defects	March 2007
	Expanded indication for spinal fusion procedures in skeletally mature patients with degenerative disc disease at 1 level from L4 to S1 Expanded for acute, indication open tibial shaft fractures stabilized with nail fixation	October 2009
INFUSE™ Bone Graft/LT-CAGE™ Lumbar Tapered Fusion Device	Indicated for spinal fusion procedures in skeletally mature patients with degenerative disc disease at 1 level from L4 to S1 Up to grade 1 spondylolisthesis at the involved level Implantation via anterior open or anterior laparoscopic approach	July 2002
	Extension of device use from L2 to S1 May be used with retrolisthesis	July 2004
	Indicated for acute, open tibial shaft fractures stabilized with nail fixation Alternative to autogenous bone graft for sinus augmentations For localized alveolar ridge augmentations in extraction socket defects	October 2009
INFUSE™ Bone Graft/Medtronic Interbody Fusion Device	Indicated for spinal fusion procedures in skeletally mature patients with degenerative disc disease at one level from L2-S1, who may also have up to Grade I spondylolisthesis or Grade I retrolisthesis at the involved level	December 2015

**Table 3:** Summary of preclinical studies applying EVs in immunoregulation, osteoclastogenesis, osteoblastogenesis, and angiogenesis. ^+^ Bone Marrow Mesenchymal Stem Cell-Derived Extracellular Vesicles.

Author	Parent Cell	EV Dose	Scaffold Carrier	Cargo; Pathway	Growth Factor, Protein, Gene	Model	Outcome
Takeuchi et al. [111]	hBMMSCs	30 μg	Atelocollagen sponge	miRNA undefined; unspecified	↑ VEGF, ANG1, ANG2, COLI, OCN, Runx2	Wistar rat calvarial defect	In vitro: anti-VEGF antibody decreased expression of osteogenic and angiogenic-related genes; EVs promoted hMSC migration In vivo: anti-VEGF antibody impaired bone formation
Zhang et al. [117]	Rat BMMSCs	100 μL (10^10^ particles)	Local injection	BMP; BMP-2/Smad1/Runx2 and HIF1-1α/VEGF pathways	↑ OCN, OPN, OGN, BMP2, Smad1, Runx2	Wistar rat femoral nonunion	In vitro: BMMSC-EVs ^+^ promoted proliferation and migration of HUVECs and osteoblast precursors In vivo: BMMSC-EVs ^+^ enhanced osteogenesis, angiogenesis, and fracture healing
Zhang et al. [118]	hBMMSCs	25 μg/mL	Local injection	miR-935; inhibition of STAT1	↑ Runx2, ATF4	Sprague–Dawley ovariectomized, osteoporotic rats	In vitro: increased ALP activity, enhanced levels of Runx2 and ATF4, enhanced osteoblast proliferation and differentiation In vivo: increased BMD, BV/TV, TbN, Tb.Th
Li et al. [100]	hADSCs	25 μg/mL	PLGA/pDA	miR-218; unspecified	↑ Runx2, ALP, COL1a1	Murine critical-sized calvarial defect	In vitro: enhanced expression of osteoblastogenesis-related genes In vivo: significantly more new bone formation and recruitment of host MSCs
Li et al. [119]	hADSCs	0.8 mg/mL	GNP hydrogel	miR-451a; unspecified	↑ M2 marker (CD206) ↓ M1 marker (iNOS)	Sprague–Dawley rat calvarial defect	In vitro: miR-451a promotes the polarization of macrophage phenotypes through the inhibition of MIF In vivo: immunoregulated bone microenvironment, promoted osteogenesis
Zhang et al. [120]	hUCMSCs	100 μL/mL	HyStem-HP hydrogel	HIF-1α, VEGF; unspecified	↑ HIF-1α, VEGF, OCN, COL1a1	Rat femoral fracture	In vitro: Upregulation of osteogenic- and angiogenic-related gene expression levels In vivo: promoted angiogenesis and fracture healing through the proliferation of HUVECs
Qi et al. [121]	hiPSC-MSCs	100 µg	β-TCP	unspecified	↑ OPN, COL1, Runx2	Sprague–Dawley ovariectomized rats with calvarial defect	In vitro: increased ALP activity and expression levels of osteoblast-related genes and increased proliferation of rBMSCs In vivo: enhanced BV/TV and angiogenesis in a dose-dependent manner
Cui et al. [122]	MC3T3-E1	100 µg	----	miR-1192, miR-680, miR-302a; Wnt pathway	↑ Runx2, ALP, β-catenin ↓ Axin1	Murine bone marrow-derived stromal cell line (ST2)	In vitro: increased osteoblast differentiation and matrix mineralization
Uenaka et al. [123]	MC3T3-E1, Mature osteoblasts	1–5 × 10^9^ particles	Gelatin-hydrogel sheet	miR-143-3p; targeting of *Cbfb*	↑ Rankl ↓ Runx2, Sp7	Murine critical-sized calvarial defect	In vitro: Inhibition of osteoblast differentiation and promotion of osteoclastogenesis through the suppression of osteoblastic gene expression In vivo: inhibition of bone repair and promotion of bone resorption
Eichholz et al. [124]	MLO-Y4 osteocyte-like cells	1 μg	----	Annexin A5, Histone H4; inhibition of RANKL-RANK	↑ (CM-F): Histone H4, COX2, OCN, OPN, Runx2, OSX, ALP	hMSCs	In vitro: CM-F treatment groups enhanced osteogenesis, osteoblastogenesis
Lv et al. [125]	MLO-Y4 osteocyte-like cells	10 μL	----	miR181b-5p; PTEN/AKT pathway	↑ ALP, BMP2, Runx2	hPDLSC	In vitro: promoted osteogenic proliferation and differentiation in mechanically strained MLO-Y4 cells

↑, increased; ↓, decreased; hBMMSCs, human bone marrow mesenchymal stem cell; miRNA, microRNA; VEGF, vascular endothelial growth factor; ANG1, angiopoietin 1; ANG2, angiopoietin 2; COL1, type 1 collagen; OCN, osteocalcin; Runx2, runt-related transcription factor 2; HUVEC, human umbilical vein endothelial cells; BMP-2, bone morphogenetic protein 2; HIF1-1α, hypoxia-inducible factor 1-alpha; OPN, osteopontin; OGN, osteoglycin; STAT1, signal transducer and activator of transcription 1; ATF4, activating transcription factor 4; BMD, bone mineral density; BV/TV, bone volume/tissue volume; TbN, average number of trabeculae per unit length; Tb.Th, mean thickness of trabeculae; hADSCs, human adipose stem cells; PLGA, poly(lactic-co-glycolic) acid; pDA, polydopamine; ALP, alkaline phosphatase; COL1a1, collagen type 1 alpha 1; GNP, gliadin nanoparticles; M2, macrophage phenotype 2; M1, macrophage phenotype 1; MIF, macrophage migration inhibitory factor; iNOS, inducible nitric oxide synthase; hUCMSCs, human umbilical cord mesenchymal stem cells; hiPSC, human-induced pluripotent stem cells; β-TCP, beta-tricalcium phosphate; Rankl, receptor activator of nuclear factor kappa beta; RANK, receptor activator of nuclear factor kappa beta; COX2, prostaglandin-endoperoxide synthase 2; OSX, osterix; hMSCs, human mesenchymal stem cells; CM-F, media undergone fluid shear; PTEN/AKT, phosphate and tensin homolog/protein kinase B; hPDLSC, human periodeontal ligament stem cell.

**Table 4:** Ex vivo viral vectors used to deliver BMP to treat critical-sized defects and promote spinal fusion in preclinical animal models.

Author	Vector	Cell	Carrier	Model	Results
Lieberman et al. [160]	Ad-BMP2	W-20 (murine stromal)	DBM	SCID Mouse 8 mm Femoral Defect	Radiographic healing at 8 wks. Histologic demonstration of lamellar bone formation
Lieberman et al. [20]	Ad-BMP2	Rat Bone Marrow Cells	DBM	Lewis Rat 8 mm Femoral Defect	Radiographic healing at 8 wks with course trabecular bone with remodeling. Equivalent mechanics between operated and non-operated femurs that healed
Dumont et al. [176]	Ad-BMP9	Human Mesenchymal Stem Cells	----	Athymic Nude Rat Lumbar Fusion	MicroCT evidence of ectopic bone formation with histologic demonstration of fusion with posterior spinal elements
Wang et al. [179]	Ad-BMP2	Rat Bone Marrow Cells	DBM or collagen sponge	Lewis Rat Lumbar Fusion	Radiograph, histologic, and mechanical testing demonstrate spinal fusion in both carriers
Hidaka et al. [175]	Ad-BMP7	Rat Bone Marrow Cells	Allogeneic Allograft	Lewis Rat Lumbar Fusion	Radiograph, histologic, and mechanical testing demonstrate spinal fusion
Lee et al. [180]	Ad-BMP2	Human Myocytes	Collagen Matrix	SCID Mouse 5 mm Calvarial Defect	Bridging bone appears at 2 wks postoperatively with significant healing and periosteum at 4 wks
Feeley et al. [161]	LV-BMP2 or Ad-BMP2	Rat Bone Marrow Cells	Collagen Sponge	SCID Mouse 4 mm Radial Defect	LV-BMP2 continued BMP2 production at 12 wks compared to only 4 wks for Ad-BMP2, with radiographic and histologic healing for both vectors
Virk et al. [162]	LV-BMP2 or Ad-BMP2	Rat Bone Marrow Cells	Calcium Phosphate plus Type I Collagen	Lewis Rat 8 mm Femoral Defect	Higher rates of healing on radiographs and microCT with improved mechanical properties for LV-BMP2 vs. Ad-BMP2
Virk et al. [152]	LV-BMP2	“Same-Day” Rat Bone Marrow Cells vs. Traditional	Calcium Phosphate plus Type I Collagen	Lewis Rat 8 mm Femoral Defect	“Same-Day” ex vivo technique resulting in faster rates of healing with increased bone formation and improved biomechanics compared to traditional methods
Alluri et al. [181]	LV-BMP2	Rat Bone Marrow Cells	3D-printed Tricalcium Phosphate Scaffold	Lewis Rat 6 mm Femoral Defect	Radiographic healing with histology demonstrating trabecular bone circumferentially
Miyazaki et al. [173]	LV-BMP2	Rat Bone Marrow Cells	Collagen Carrier	Lewis Rat Lumbar Fusion	Radiographic, microCT, and histologic evidence of healing with resorption of collagen sponge
Miyazaki et al. [172]	LV-BMP2 or Ad-BMP2	Rat Bone Marrow Cell	Collagen Carrier	Lewis Rat Lumbar Fusion	Improved spinal fusion with LV-BMP2 seen on radiographs, microCT, and histology vs. Ad-BMP2
Miyazaki et al. [182]	Ad-BMP2	Human Bone Marrow or Adipose Stem Cells	Collagen Carrier	Athymic Nude Rat Lumbar Fusion	Equivalent fusion on radiographics, microCT, histology, and mechanical testing between bone marrow and stem cell groups
Vakhshori et al. [151]	LV-BMP2	Human Adipose Stem Cell	Tricalcium phosphate/Hydroxyapatite	Athymic Nude Rat 6 mm Femoral Defect	Equivalent radiograph, microCT, histological, and biomechanical testing compared to rhBMP
Kang et al. [183]	LV-BMP2	Rat Bone Marrow Cells	3D Printed Hyperelastic bone	Lewis Rat 6 mm Femoral Defect	Radiographic and histologic evidence of healing with bony ingrowth on scaffold

Ad-BMP2, adenovirus vector expressing a bone morphogenetic protein-2; DBM, demineralized bone matrix; SCID, severe combined immunodeficiency; LV-BMP2, lentivirus vector expressing a bone morphogenetic protein-2; rhBMP, recombinant human bone morphogenetic protein.

**Table 5:** In vivo viral vectors used to deliver BMP in preclinical animal models.

Author	Vector	Model	Results
Rundle et al. [184]	MLV-BMP2/BMP4 hybrid	Sprague–Dawley Femur Fracture	Significant callous formation early post-injection; however, mostly found to be extra-periosteal. Histology failed to demonstrate significant differences in remodeling compared to controls.
Baltzer et al. [185]	AV-BMP2	New Zealand White Rabbit Femoral Defect	Defects were 75% restored after 7 wks and healed after 12 wks in the experimental group only. Histology demonstrated a bridging callus present at 8 wks post-injection.
Helm et al. [178]	AV-BMP9	Athymic Nude Rat Lumbar Fusion	16 wks post-injection microCT demonstrated fusion mass in direct contact with posterior spinal elements without canal compromise. Histological evidence of lamellar bone with marrow cavities developed.
Alden et al. [174]	AV-BMP2	Athymic Nude Rat Lumbar Fusion	12 wks post-injection microCT demonstrated fusion mass in direct contact with posterior spinal elements. Sharp borders observed on histology but no adverse reaction in surrounding paraspinal musculature.
Laurent et al. [177]	AV-BMP6	New Zealand White Rabbit Lumbar Fusion	14 wks post-injection microCT demonstrated fusion mass in direct contact with posterior spinal elements. Histological evidence of bony bridging between transverse processes.

MLV-BMP2/BMP4, murine leukemia virus retroviral vector expressing bone morphogenetic protein 2 and 4. AV-BMP, adenovirus vector expressing bone morphogenetic protein.

## Data Availability

No new data was reported in this review article. Cited studies can be accessed from the references already included.
